# Acoustic Telemetry Validates a Citizen Science Approach for Monitoring Sharks on Coral Reefs

**DOI:** 10.1371/journal.pone.0095565

**Published:** 2014-04-23

**Authors:** Gabriel M. S. Vianna, Mark G. Meekan, Tova H. Bornovski, Jessica J. Meeuwig

**Affiliations:** 1 School of Animal Biology and The UWA Oceans Institute, The University of Western Australia, Perth, Western Australia, Australia; 2 Australian Institute of Marine Science, Perth, Western Australia, Australia; 3 Micronesian Shark Foundation, Koror, Palau; Università degli Studi di Napoli Federico II, Italy

## Abstract

Citizen science is promoted as a simple and cost-effective alternative to traditional approaches for the monitoring of populations of marine megafauna. However, the reliability of datasets collected by these initiatives often remains poorly quantified. We compared datasets of shark counts collected by professional dive guides with acoustic telemetry data from tagged sharks collected at the same coral reef sites over a period of five years. There was a strong correlation between the number of grey reef sharks (*Carcharhinus amblyrhynchos*) observed by dive guides and the telemetry data at both daily and monthly intervals, suggesting that variation in relative abundance of sharks was detectable in datasets collected by dive guides in a similar manner to data derived from telemetry at these time scales. There was no correlation between the number or mean depth of sharks recorded by telemetry and the presence of tourist divers, suggesting that the behaviour of sharks was not affected by the presence of divers during our study. Data recorded by dive guides showed that current strength and temperature were important drivers of the relative abundance of sharks at monitored sites. Our study validates the use of datasets of shark abundance collected by professional dive guides in frequently-visited dive sites in Palau, and supports the participation of experienced recreational divers as contributors to long-term monitoring programs of shark populations.

## Introduction

Many shark species are experiencing unsustainable rates of mortality due to fishing, a phenomenon that is driving population declines globally [1]. Despite this emerging crisis, our knowledge of the distribution, abundance and ecology of many species is generally poor. In 2014, an assessment of the extinction risk of 1,041 species of elasmobranchs concluded that almost half (487 species) were categorised as “Data Deficient”, meaning that a lack of information prevented any firm conclusions being drawn on their population status and trajectories [2]. This has occurred at a time when there is increasing evidence of the importance of sharks as top-down regulators of the structure and function of marine ecosystems [3] and recognition of their current and potential value as a non-consumptive resource that supports local economies through ecotourism [4].

The assessment and monitoring of shark populations through fishery-independent techniques presents considerable challenges due to the naturally low population densities and relatively large home ranges common to most species [5], [6]. The large scale (tens to hundreds of km) and long-term (years to decades) monitoring programs that can be required to document the status of populations are thus expensive, particularly if they involve in-water activities such as SCUBA diving. For this reason, implementing these initiatives is often beyond the means of governments of developing countries or organisations with interests in the conservation of sharks. Thus, there is an urgent need for the creation and adoption of simple, standardised and low-cost methods for monitoring shark populations [7].

Data collected by the public can provide a cost-effective means of monitoring populations of wild animals [8]. Such “citizen science” initiatives are growing in popularity as alternatives to conventional scientific sampling as they offer the opportunity to gather large datasets at reduced cost [9], [10]. In the marine environment, this approach is particularly useful for the study of conspicuous animals and megafauna inhabiting coastal areas and coral reefs, where data obtained from the public are relatively easy to collate. This approach has been used to describe the distribution and ecology of many species, including green and hawksbill turtles (*Chelonia mydas* and *Eretmochelys imbricata*), minke whales (*Balaenoptera acutorostrata*) and manta rays (*Manta alfredi*) [11]–[13]. Sharks have also been the target of many of these initiatives, with projects based on data collected by recreational snorkelers and divers used to investigate patterns in distribution, demographics, abundance, habitat use, movement and the effects of environmental and anthropogenic factors [7], [14]–[19]. Structured programs where data are gathered by recreational participants have also been used to provide baseline data and monitor spatial and temporal trends in abundance, which can then be used to design and assess the efficiency of conservation measures [13], [15].

Recreational divers and snorkelers have been used to collect data on elasmobranchs in two principal ways: firstly, by recording counts of animals seen during a dive [7], [16], [20] and secondly, by taking identification photos that can then be used in mark-recapture modelling to estimate trends in abundance and demography [17], [21]. The latter approach focuses on those species that have distinctive patterning or scars that allow animals to be identified individually, such as whale sharks (*Rhincodon typus*), manta rays and white sharks (*Carcharodon carcharias*), but is unsuitable for the many reef and pelagic sharks that generally lack any persistent features that might be used to distinguish individuals. For these species, counts by divers provide one of the simplest means to monitor numbers.

Traditional approaches to underwater visual surveys involve standardized techniques that focus on quantifying the area sampled and the abundance and length of individuals within the sample space [5], [22]. Such rigorous protocols are a feature of science-based diving programs, but are not necessarily applied during recreational diving. For this reason, datasets of counts collected by recreational divers do not usually generate the data necessary for calculations of total abundance, density and biomass as area-based metrics [5], [23]. It is also recognised that other issues may potentially compromise the quality of recreational datasets, such as rounding bias, misidentification and inflation of estimates [7], [9], [20]. Although simulation and comparative studies have suggested that recreational divers may indeed be able to report shark numbers in an accurate and reliable manner [7], [23], there is a need for independent validation of this approach in the field.

Acoustic telemetry can provide a means to address these issues and validate datasets generated by citizen science programmes. Arrays of acoustic receivers are now commonplace in many coastal marine environments that are inhabited by marine megafauna such as sharks [24]. Acoustic tags can be deployed on animals without causing modification of behaviour and will report their presence whenever they are within range of the array [24]. The presence/absence data generated by these tags are commonly used to monitor the attendance of individuals tagged at the monitored sites [24], and provide an index of relative abundance that can be used to identify trends in the populations over time. In places where citizen science initiatives and arrays overlap, acoustic telemetry can be used to assess the validity of citizen science datasets.

In our study, we compared datasets of shark relative abundance collected by professional dive guides with tagging data generated by passive acoustic telemetry at the same sites on coral reefs in Palau, Micronesia. We aimed to determine if the observations reported by dive guides produced comparable estimates of relative abundances and temporal patterns in numbers of sharks as those obtained from presence/absence data derived from acoustic tagging and monitoring. We also used the telemetry data to investigate the effect of the presence of tourist divers (i.e. observer effect) on the relative abundance and depth use by sharks. Finally, we analysed the citizen science data in order to identify environmental correlates of patterns of relative abundance of sharks at dive sites.

## Methods

### Ethics Statement

This project was conducted under the Republic of Palau Marine Research Permit no. RE-09-26 and the Koror State Marine Research Permit no. 10–204. Shark tagging in 2011 was also conducted under UWA animal ethics permit no. RA/3/100/975, in adherence to provisions contained within the Australian Code of Practice for the Care and Use of Animals for Scientific Purposes. Participants of the shark counts were aware of the use of these data for research and provided written consent for the use of the data collected. This project has been assessed as exempt from ethics review by the Human Research Ethics Office of the University of Western Australia (protocol code: RA/4/1/6457).

### Study Area

Palau supports a dive industry consisting of approximately 20 tourism businesses that use mainly small speed boats to transport tourists to local reef sites for two to three dives per day. In 2010, it was estimated that approximately 41,000 tourists visited Palau to engage in dive activities, of which approximately 8,600 visited the country specifically to dive with sharks [4]. Most of the popular dive sites are on the southwest (leeward) area of the barrier reef that surrounds Babeldaob, the main island of Palau (7°N, 134°W). This area encompasses dive sites that vary in topography from relatively sheltered sand flats and coral gardens to steep walls and promontories that project into oceanic waters on the outer reef slope [25]. Diving occurs in a variety of habitats including sandy channels and caverns, however the main drawcard for divers visiting Palau are the “drop-off” dives on the steep reef slope exposed to the open ocean, where there are usually moderate to strong tidal currents. These dives sites are characterised by high visibility (>30 m) and a rich diversity of marine life with high abundances of large pelagic species. Many of these dive sites host aggregations of reef sharks, which are composed mainly of resident grey reef (*Carcharhinus amblyrhynchos*) and whitetip reef sharks (*Triaenodon obesus*) [4], [26]. Dives at these sites are typically conducted during periods of relatively high tidal currents when sharks swim just off the edge of the slope. Divers enter the water up-current and, on arrival at the aggregation sites, attach themselves by a hook and line to the reef crest so that they can remain stationary to view sharks and large fish passing along the drop-off [4]. Once safety time limits at these depths (typically between 12–25 m) are attained, the divers release hooks and lines and drift along the reef crest making a slow ascent to the surface. For safety reasons, the routes and durations of these dives are similar through time (GMSV. and MGM. pers. obs.).

### Data Collection

Our dataset consisted of counts of sharks sighted during dives by dive guides who worked as employees of a dive tourism business. Standard questionnaires were completed after the day trips by dive guides from October 2007 to November 2012. A total of 62 dive guides recorded information for 2,360 dives at 52 dive sites in Palau. Each questionnaire contained information on the dive site visited, date, species and counts of individual sharks sighted by the guide. Dive guides estimated the depth of the divers during the sightings, current strength (0- no current; 1- weak; 2- moderate; 3- strong current), visibility (in meters), and recorded dive time and number of tourist divers in the group. Each completed questionnaire provided observations from a single dive guide.

Most of the dive guides participating in the study were local residents familiar with the identification reef shark species in Palau. Guides were instructed to report the total number of individual sharks of each species observed during the entire dive. We also instructed the dive guides to be conservative with counts, observing features that could permit individual identification (e.g. pigment patterns, marks and scars) and reduce the potential for repeated counts. An office staff member was responsible for the administration and management of the survey, encouraging dive guides to return questionnaires regularly. This staff member was also trained to enter data and maintain the dataset. To promote engagement and consistency in data collection, we also established an annual event to provide feedback to the participants, where the dive guides who collected data regularly would receive small rewards. During these events, the researchers involved in the project would also provide lectures to the dive community, where updates on relevant issues and results obtained from the dataset were presented.

**Figure 1:**
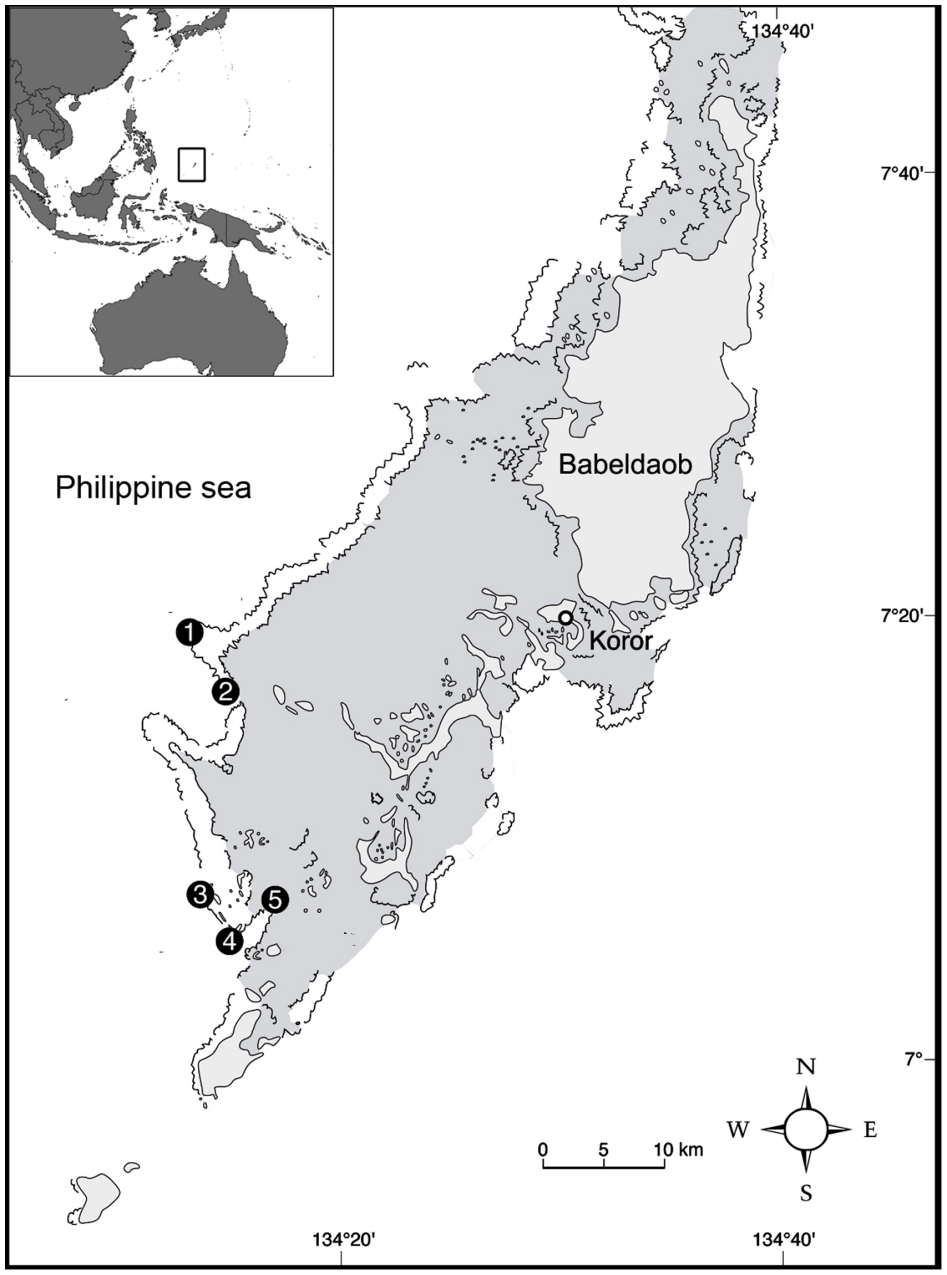
Study area in Palau. Numbers indicate location of dive sites monitored: 1) Siaes Corner, 2) Ulong Channel, 3) Blue Corner, 4) New Drop-off and 5) German Channel. Grey shade represents lagoonal area, light grey indicates islands. Numbers one to four also indicate the location of acoustic receivers.

We used passive acoustic telemetry to monitor the attendance of resident grey reef sharks at four key dive sites (Blue Corner, Siaes Corner, Ulong Channel and New Drop-off), known to host predictable aggregations of sharks (Figure 1). An array of four Vemco VR2w acoustic receivers monitored sharks tagged with acoustic transmitters from November 2008 to December 2012, a period that overlapped the dive guide records of shark counts. The receivers were deployed at depths between 25 and 40 m on the barrier reef drop-off or slope and recorded the presence of tagged sharks within 200 m of the receiver location [26]. We internally tagged 39 grey reef sharks (38 females, 1 male) with Vemco V16 coded tags with battery life ranging between three and a half and ten years. Ten of these tags were also fitted with pressure sensors, which provided a record of depth of the tagged sharks (for full description see [26]). Temperature loggers were deployed near the dive sites at 15 m depth and provided records of daily temperature from January 2009 to March 2012. We used the number of sharks detected by the receivers as an index of the relative abundance of the tagged sharks at the monitored sites.

### Data Analysis

We limited our analysis to data from experienced dive guides, defined as guides who participated for at least three of the five years during which the study was undertaken, who had returned a minimum of 100 questionnaires. In order to obtain a reasonable representation of questionnaires for each calendar month, our analysis focused on the dive sites that also yielded a minimum of 100 questionnaires. We estimated the daily relative abundance (no. day^−1^) of sharks observed by dive guides as the mean value of all dives in a given day at the same site. Given daily variation in diving activities, we also calculated a mean daily abundance for each calendar week.

We calculated the frequency of occurrence of each species as the proportion of days reported when a given species was sighted by dive guides. Our statistical analyses focused on grey reef and whitetip reef sharks, the two most abundant species recorded by dive guides at our study sites. We first used linear regression to compare the daily relative abundance of grey reef sharks observed by dive guides and the number of individually tagged sharks recorded by receivers at the same site on the same day. This analysis allowed us to determine whether there were correlations between diver counts and the number of sharks tagged attending the array. Our dataset only included days when sharks were detected by receivers on a minimum of two occasions.

We also used linear regression to investigate the effects of the presence of divers on the behaviour of sharks at the study sites. For this analysis, we regressed the number of tagged grey reef sharks present at a given site and day against the number of tourist divers reported to be in the water during the corresponding day. Multiple dives reported on the same day at a given site were treated as separate samples unless occurring simultaneously (in the same hour), in which case we summed the number of tourist divers reported by each guide as a measure of the total potential influence on the sharks. We also used linear regression to analyse the relationship between the mean depth of the tagged sharks and 1) the number of tourist divers reported to be in the water during a dive and 2) the mean depth of these divers.

We used circular regression [27] to relate monthly patterns in mean daily relative abundance of grey reef sharks observed by dive guides with telemetry records. We used a *t*-test for slopes to compare the regressions of monthly observations by dive guides with the mean number of grey reef sharks detected using telemetry. Monthly estimates of relative abundance observed by dive guides and numbers of sharks detected by telemetry were calculated by averaging the daily values across a given month. For the regression, we included only telemetry values that had two or more corresponding observations by dive guides. The explanatory variable “month” was sine-transformed to account for cyclical variation in abundance of sharks. We also fitted circular regressions to investigate patterns of seasonality in the mean monthly relative abundance of sharks observed by dive guides at the selected study sites. This analysis was performed for all sharks combined but also separately for grey reef and whitetip reef sharks.

We used multiple linear regression to examine the influence of environmental factors on the relative abundance of sharks observed by dive guides. Our response variable was the log-transformed daily abundance of sharks (i.e. all sharks combined, grey reef, and whitetip reef sharks) averaged per week. Explanatory variables included in the models were: year, current strength, temperature, visibility, number of tourist divers and moon phase. We applied the Akaike’s Information Criterion (AIC) test statistics including AIC, _Δ_AIC (difference between AIC of a given model and best fitted model) and _w_AIC (weighted AIC) to select the models with best fit [28]. Since the order of inclusion of variables influences the AIC model selection process, we analysed the correlation coefficients of explanatory and response variables and used the function Regsubsets of the “leaps” R-package [29] to determine the order of inclusion of the variables in building the models. We validated our models by inspecting the residuals for patterns indicating likely violation of assumptions and fitted correlograms to visually inspect our dataset for auto-correlation. This analysis was performed for the data collected at Blue Corner, the site that yielded the largest number of weekly records (*n* = 148) from the dive guides.

All analyses used the R statistical software [30] and all summary metrics were reported as mean values and standard errors (±SE).

## Results

### Dive Guide Datasets

Our final dataset, filtered to only include records of the most frequently visited dive sites and observations of selected guides (those returning more than 100 questionnaires), consisted of data for 1,252 dives (53%) that were collected by 24 dive guides (39%) at five dive sites (10%) (Blue Corner, Siaes Corner, New Drop-off, Ulong Channel and German Channel) over a period of five years. These dive sites are known to be aggregations or hotspots of charismatic megafauna, including reef sharks. The total number of dives at each site varied from 118 at Siaes Corner to 388 at Blue Corner, with an overall mean dive time of 57±0.22 min and a mean dive depth of 16±0.18 m (Table 1).

**Table 1:** Descriptive metrics of dive guide observations and telemetry data at the monitored sites in Palau.

	Blue Corner					Ulong Channel						Siaes Corner						New Drop-off						German Channel						Total					
Metrics	Mean	Min	Max	SD	*n*		Mean	Min	Max	SD	*n*		Mean	Min	Max	SD	*n*		Mean	Min	Max	SD	*n*		Mean	Min	Max	SD	n		Mean	Min	Max	SD	*n*
Number of dives	388	–	–	–	–		223	–	–	–	–		118	–	–	–	–		138	–	–	–	–		385	–	–	–	–		250.4	118	388	130.4	5
Estimated areaof dive site (m^2^)	17000	–	–	–	–		27000	–	–	–	–		19000	–	–	–	–		22000	–	–	–	–		10000	–	–	–	–		19000	10000	27000	6285	6
Dives per month	7.9	1	31	5.3	48		4.8	1	18	3.4	45		2.8	1	7	1.6	41		3.3	1	13	2.5	40		7.9	1	37	6.0	47		5.7	1	48	4.6	48
Dive time (min)	56	30	70	6.4	354		60	25	88	7.6	206		57	20	68	6.3	108		57	45	85	6.5	123		57	30	80	6.8	336		57.4	20	88	6.9	1127
Divers depth	16.3	5	35	5.1	258		15.4	5	45	4.7	158		18	10	25	5.4	86		15.4	5	30	5.3	87		15.7	5	30	5.1	281		16.2	5	45	5.1	870
Divers per dive	17.5	10	40	7.5	354		16	10	40	6.7	204		15.5	10	30	6.6	110		13.9	10	30	5.2	123		19.4	10	40	8.3	334		17.2	10	40	7.5	1125
Days withtelemetry data	185	–	–	–	–		122	–	–	–	–		67	–	–	–	–		32	–	–	–	–		–	–	–	–	–		101.5	32	185	66.9	4
Sharks detecteddaily (telemetry)	7.2	2	19	2.9	185		6.9	3	13	2.6	122		2.7	1	8	1.3	67		3.2	2	7	1.0	32		–	–	–	–	–		6.0	1	19	3.1	406

SD: standard deviation, *n*: sample size.

Dive guides reported seeing sharks during all dives at the selected sites. Grey reef and whitetip reef sharks were the species most commonly observed, with a frequency of occurrence of 86% and 83% and mean relative abundance per dive of 10.1±0.3 and 5.3±0.1 respectively (Table 2). Blacktip reef sharks (*Carcharhinus melanopterus*) were also sighted frequently (14% of dives) but with relative abundance of 0.3±0.1 sharks per dive. The other species of observed sharks were recorded very infrequently (2% of dives) and in low numbers (0.02 sharks per dive) (Table 2). Shark relative abundances at Blue Corner and Ulong Channel were higher than at other sites with mean values of 20.3±0.6 and 19.0±0.7 per dive respectively, while lower relative abundance was recorded at German Channel with mean value of 11.1±0.4 sharks per dive (Table 2).

**Table 2:** Frequency of occurrence (FO) and mean daily relative abundance of sharks observed by dive guides (Abund) and standard error (SE) of most common species of sharks at the monitored sites in Palau.

	Blue Corner		Ulong Channel		Siaes Corner		New Drop-off		German Channel		Overall	
Species	FO (%)	Abund	FO (%)	Abund	FO (%)	Abund	FO (%)	Abund	FO (%)	Abund	FO (%)	Abund
All sharks	100	20.3 (0.6)	100	19.0 (0.7)	100	13.8 (0.9)	100	11.5 (0.6)	100	11.1 (0.4)	100	15.6 (0.3)
Grey reef shark	86	12.4 (0.5)	89	13.4 (0.6)	94	10.9 (0.9)	87	6.9 (0.6)	83	6.8 (0.3)	86	10.1 (0.3)
Whitetip reef shark	86	7.7 (0.3)	86	5.5 (0.3)	76	2.7 (0.3)	88	4.3 (0.3)	81	4 (0.2)	83	5.3 (0.1)
Blacktip reef shark	13	0.3 (0.1)	9	0.2 (0.2)	8	0.2 (0.1)	22	0.4 (0.2)	17	0.25 (0.1)	14	0.3 (0.1)
Other species^a^	1	0.01 (0.0)	1	0.01 (0.1)	0	0	1	0.01 (0.0)	4	0.04 (0.1)	2	0.02 (0.0)

a Other species include hammerhead, zebra, bull, nurse, and lemon sharks.

### Integration of Dive Guide and Telemetry Data

Paired sets of abundance estimates provided from dive guides and acoustic detections were available for 406 dives (Table 1). For the dives when telemetry data were available, the number of sharks detected by acoustic receivers at a given site varied from one to 19 with a mean of six grey reef sharks detected per day (±0.02).

**Figure 2:**
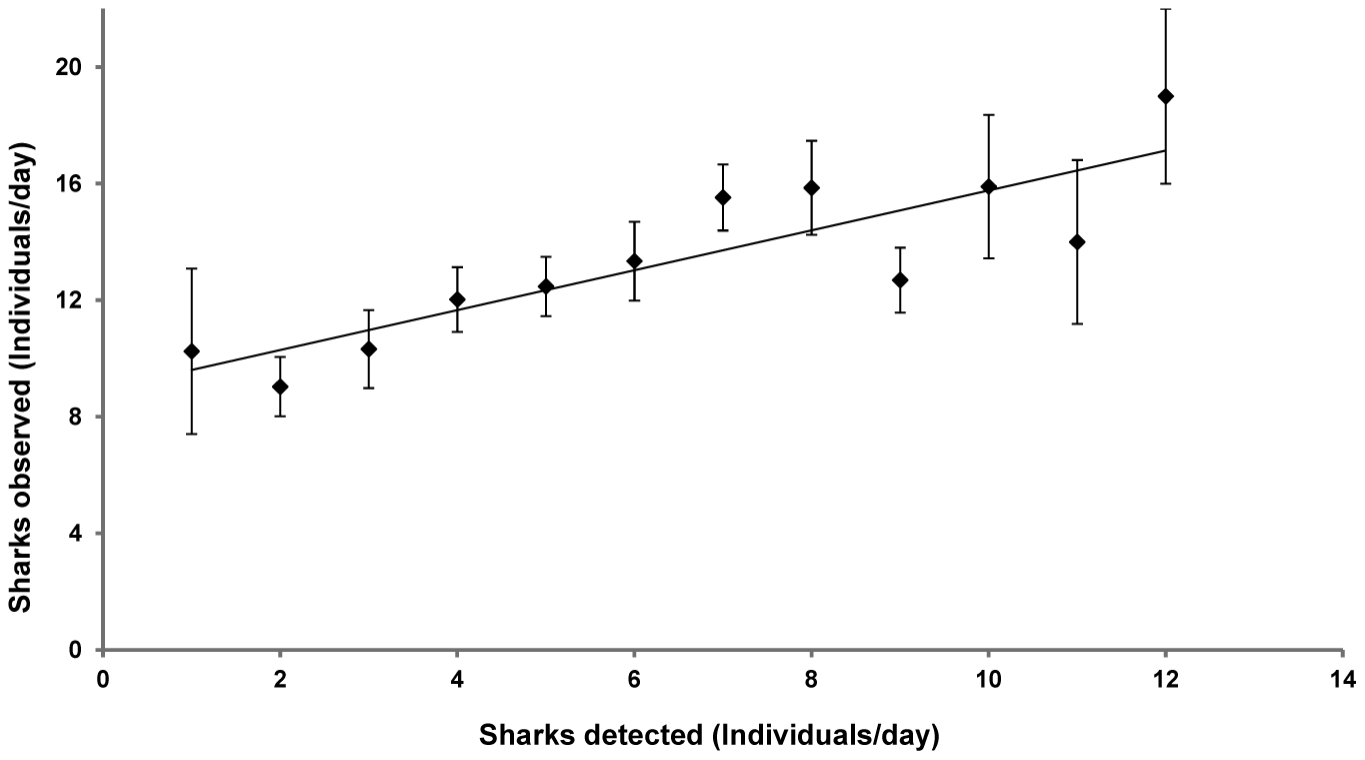
Relationship between daily underwater observations and acoustic telemetry of grey reef sharks in Palau. Mean daily relative abundance of grey reef sharks observed by dive guides as a function of daily number of sharks detected by telemetry. Error bars indicate SE. y = 0.68x+8.92, R^2^ = 0.74.

The regression analysis indicated a significant and strong relationship (*R^2^* = 0.74, *p*<0.001) between the mean daily relative abundance of grey reef sharks observed by dive guides and the number of tagged individuals detected by telemetry (Figure 2). Lowest relative abundance (10 sharks per dive) was observed on days when the number of sharks detected by telemetry was also low (1–3 sharks per day). An increase in the number of sightings by dive guides corresponded to increased numbers of tagged sharks detected acoustically. The highest relative abundance of 19 grey reef sharks was observed by guides when a maximum of 12 tagged sharks were detected on acoustic receivers.

**Figure 3:**
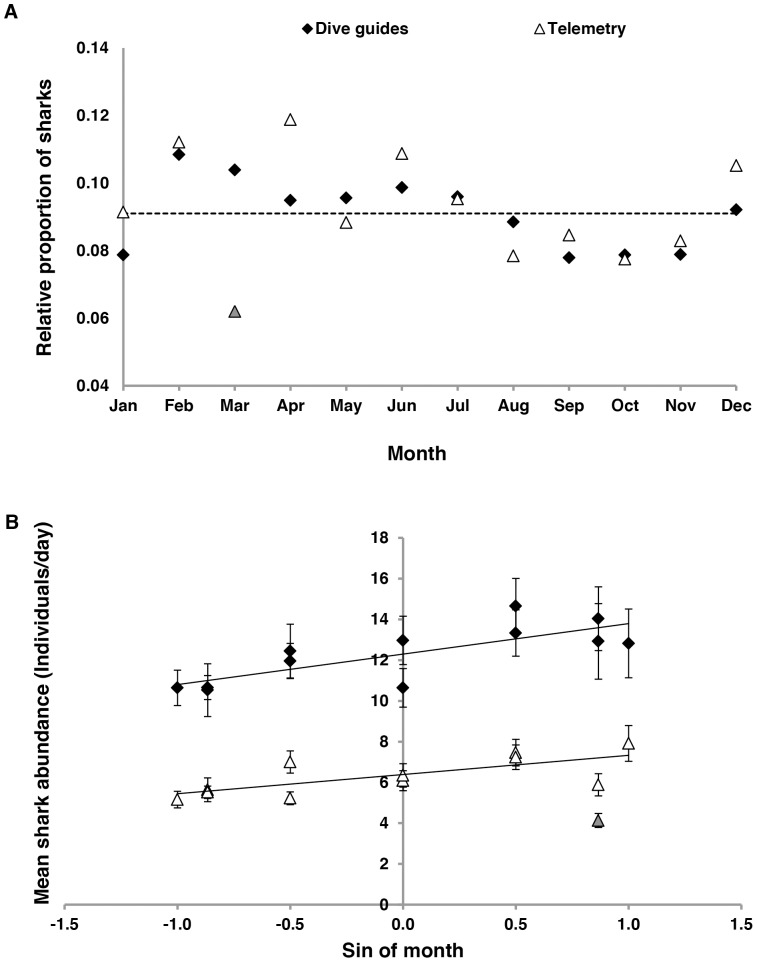
Monthly patterns of abundance of grey reef sharks at monitored sites in Palau. A) Proportion of sharks observed by dive guides and detected by telemetry monthly (calculated as the mean number of sharks observed daily in each month divided by the sum of mean values of all the months). Dashed line indicates the expected values in absence of seasonal variation. B) Mean monthly relative abundance of sharks as function of sine-transformed month. Error bars indicate SE. Dive guides: y = 1.49x+12.23, R^2^ = 0.60. Telemetry: y = 0.94x+6.39, R^2^ = 0.51. *Grey triangle indicates detection during low receiver performance and was not included in the analysis (see discussion).

**Figure 4:**
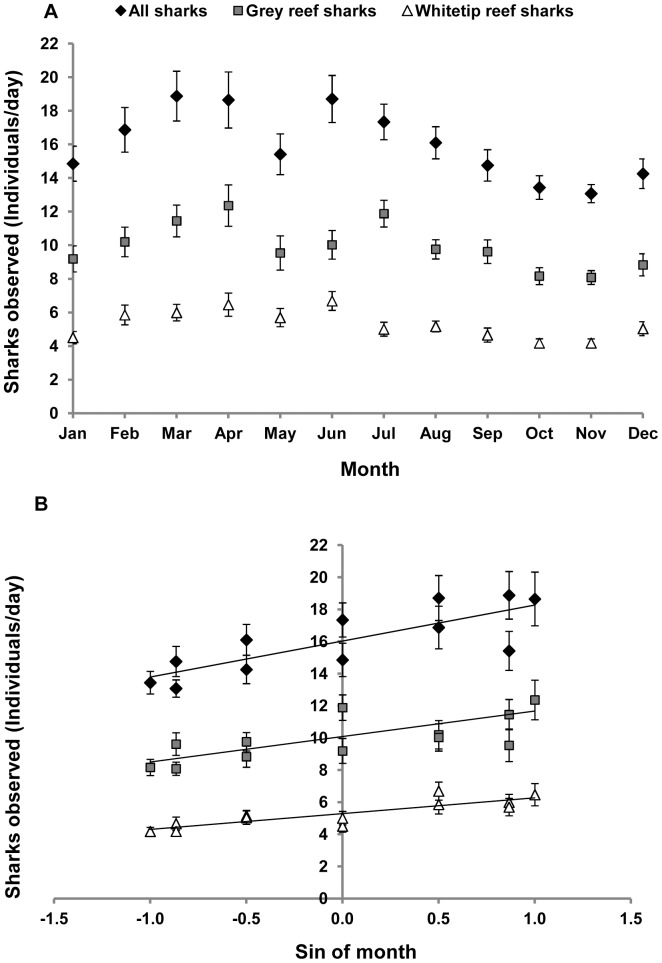
Monthly patterns of abundance of reef sharks observed by dive guides in Palau. A) Relative abundance of common species of reef sharks observed by divers at monitored sites. B) Relative abundance of common species of reef sharks observed by diver guides as a function of sine-transformed months. Error bars indicate SE. All sharks: y = 2.70x+16.30, R^2^ = 0.81, Grey reef: y = 1.59x+10.08, R^2^ = 0.64, Whitetip: y = 0.99x+5.27, R^2^ = 0.73. *May values of abundance of “All sharks” and “Grey reef sharks” not included in regressions.

Data generated by dive guides showed monthly variation in the number of grey reef sharks (Figures 3 and 4), with peaks of relative abundance occurring from February to June and lower values from August to November. This pattern was generally similar to that observed in the telemetry data (Figure 3). While there was no significant difference between the slopes of the regressions of the mean relative abundance observed by guides and telemetry detections as a function of month (*t*-test for slopes, *t* = −0.76, *p* = 0.47), there was some divergence between the two data sets during March and April (Figure 3A) when detections appeared proportionally lower than numbers observed by dive guides.

The linear regressions showed no significant relationship between the number of grey reef sharks detected by telemetry and the number of tourist divers present during the dives (*p* = 0.48). Mean values varied from 6.7±0.3 sharks detected when up 10 tourist divers were present to 7.2±1.1 when 40 or more tourist divers were present. Similarly, there were no significant relationships between the mean depth of tagged grey reef sharks and the number of tourist divers present during the dive (*p* = 0.31) or the mean depth of the tourist divers (*p* = 0.44).

### Relative Abundance of Sharks Observed by Dive Guides in Relation to Environmental Drivers

Grey reef and whitetip reef sharks were present at the monitored sites throughout the year however, there was seasonal variation in the number of individuals of both species. Lower values of monthly relative abundance occurred in October and November, with means of 8.1±0.4 for grey reef sharks and 4.2±0.2 for whitetip reef sharks. Between March and April, the relative abundance of sharks was higher with monthly means of 12.4±1.2 and 6.5±0.7 for grey reef and whitetip reef sharks respectively (Figure 4). There was a sharp decline in relative abundance of grey reef sharks in May and June to a mean of 9.4±1.2. A similar but less pronounced pattern was also observed for whitetip reef sharks with relative abundance of 5.7±1.0 in May. The circular regressions indicated that the overall seasonal patterns were statistically significant with sine (month) explaining 81% of the variation in relative abundance of all sharks (*p*<0.001), 64% of the variation in relative abundance for grey reef (*p* = 0.003) and 73% for whitetip reef sharks (*p*<0.001).

**Figure 5:**
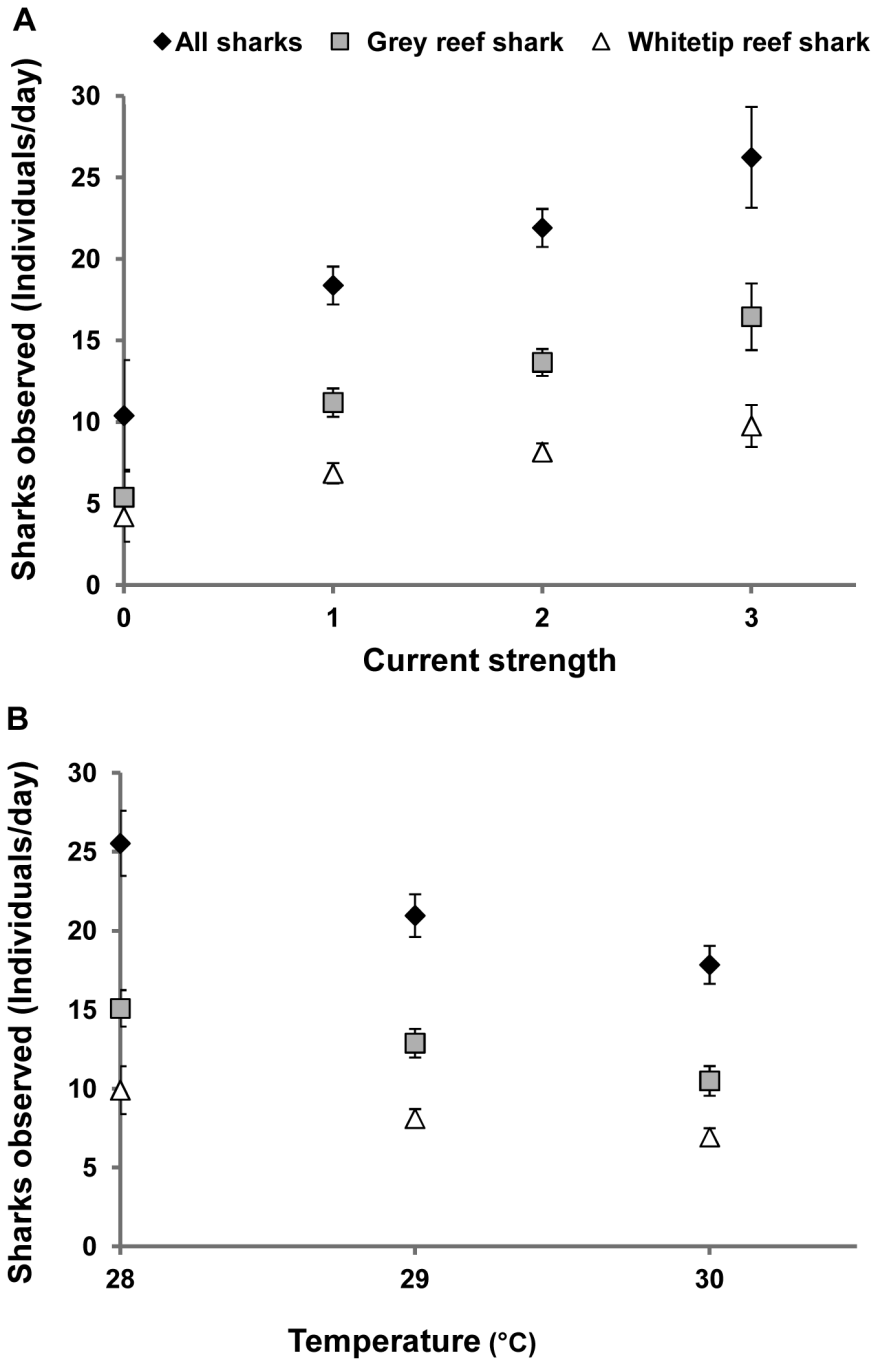
Environmental drivers of relative abundance of sharks at monitored sites in Palau. Relationship of mean daily relative abundance of sharks observed by dive guides and A) Current strength (*n* = 143 dives), and B) Temperature. (*n* = 123 dives). Error bars indicate SE.

Multiple regression indicated that current and temperature were the key environmental factors influencing the numbers of sharks recorded by dive guides (*R^2^* = 0.18, *p*<0.001; Table 3). There was a positive linear relationship between current and the relative abundance of all sharks (*R^2^* = 0.14, *p*<0.001), grey reef (*R^2^* = 0.13, *p*<0.001) and whitetip (*R^2^* = 0.07, *p* = 0.002) reef sharks, while temperature displayed a negative linear relationship with relative abundance of all sharks (*R^2^* = −0.04, *p* = 0.02) and grey reef sharks (*R^2^* = 0.05, *p* = 0.03) (Table 3, Figure 5). Year, visibility, moon phase and number of tourist divers in the water had little influence on the number of sharks sighted during the dives (Table 3).

**Table 3:** Multiple regression ranking results of the mean relative abundance of sharks observed by divers (Abund, response variable) as a function of the following explanatory variables: current strength (Cur), temperature (Temp), visibility (Vis), year, moon phase (Moon) and number of tourist divers in the water (Divers), *n* = 148 weeks.

Species	Model	df	AIC	ΔAIC	wAIC	*R^2^*	*p*-value	*F*-value
**All sharks**	Ab_DG_∼Null	2	−32.10	22.78	0.00	0	0.48	0.71
	Ab_DG_∼Cur	3	−50.25	4.64	0.05	0.14	<0.001	4.58
	Ab_DG_∼Temp	3	−36.03	18.86	0.00	0.04	0.02	−2.42
	Ab_DG_∼Vis	3	−30.43	24.46	0.00	−0.01	0.48	0.72
	Ab_DG_∼Year	3	−30.22	24.67	0.00	−0.01	0.54	−0.61
	Ab_DG_∼Moon	5	−30.22	24.67	0.00	0.01	0.28	1.28
	Ab_DG_∼Divers	3	−30.23	24.66	0.00	−0.01	0.98	0.03
	**Ab_DG_∼Cur+temp**	**4**	**−54.89**	**0.00**	**0.49**	**0.18**	**<0.001**	**14.6**
	Ab_DG_∼Cur+temp+vis	5	−53.73	1.15	0.28	0.18	<0.001	9.9
	Ab_DG_∼Cur+temp+vis+moon	8	−51.98	2.91	0.11	0.19	<0.001	5.72
	Ab_DG_∼Cur+temp+vis+moon+year	9	−50.39	4.50	0.05	0.18	<0.001	4.93
	Ab_DG_∼Cur+temp+vis+moon+divers	10	−49.98	4.90	0.04	0.18	<0.001	4.28
**Grey reef shark**	Ab_DG_∼Null	2	9.80	22.37	0.00	0	0.54	0.06
	Ab_DG_∼Cur	3	−6.24	6.33	0.01	0.13	<0.001	4.68
	Ab_DG_∼Temp	3	4.30	16.87	0.00	0.05	0.03	−2.26
	Ab_DG_∼Vis	3	11.04	23.61	0.00	−0.01	0.39	0.86
	Ab_DG_∼Year	3	11.79	24.37	0.00	−0.01	0.59	−0.54
	Ab_DG_∼Moon	5	11.90	24.48	0.00	0.01	0.29	1.28
	Ab_DG_∼Divers	3	9.36	21.94	0.00	0.01	0.21	1.26
	**Ab_DG_∼Cur+temp**	**4**	**−12.58**	**0.00**	**0.32**	**0.18**	**<0.001**	**14.35**
	Ab_DG_∼Cur+temp+moon	7	−12.14	0.43	0.26	0.20	<0.001	6.94
	Ab_DG_∼Cur+temp+moon+divers	8	−11.91	0.66	0.23	0.20	<0.001	6.10
	Ab_DG_∼Cur+temp+moon+divers+vis	9	−10.53	2.05	0.12	0.20	<0.001	5.29
	Ab_DG_∼Cur+temp+moon+divers+vis+year	10	−8.84	3.74	0.05	0.19	<0.001	4.63
**Whitetip reef shark**	Ab_DG_∼Null	2	41.28	7.63	0.01	0	0.50	0.68
	**Ab_DG_∼Cur**	**3**	**33.65**	**0.00**	**0.28**	**0.07**	**0.002**	**2.66**
	Ab_DG_∼Temp	3	41.33	7.67	0.01	0.01	0.06	−1.89
	Ab_DG_∼Vis	3	43.01	9.36	0.00	−0.01	0.85	−0.19
	Ab_DG_∼Year	3	42.66	9.01	0.00	−0.01	0.54	−0.61
	Ab_DG_∼Moon	5	42.15	8.50	0.00	0.02	0.17	1.69
	Ab_DG_∼Divers	3	41.86	8.21	0.00	0.01	0.19	−1.33
	Ab_DG_∼Cur+temp	4	33.67	0.02	0.28	0.07	0.003	5.94
	Ab_DG_∼Cur+temp+moon	7	35.34	1.68	0.12	0.08	0.009	3.24
	Ab_DG_∼Cur+temp+moon+divers	8	34.94	1.29	0.15	0.09	0.007	3.11
	Ab_DG_∼Cur+temp+moon+divers+year	9	36.46	2.80	0.07	0.09	0.01	2.72
	Ab_DG_∼Cur+temp+moon+divers+year+vis	9	36.46	2.80	0.07	0.09	0.02	2.36

Model with best fit for each analysis highlighted (bold).

AIC: Akaike's Information Criteria, _Δ_AIC: difference of the AIC of a given model to the model with best fit, _w_AIC: AIC weight, df: degrees of freedom.

## Discussion

### Citizen Science as a Means to Monitor Reef Sharks

Our analysis of data generated by dive guides suggests that citizen science initiatives can provide estimates of the relative abundance of reef sharks that are consistent with the estimates from long-term telemetry. Patterns in the relative abundance of grey reef sharks as reported by dive guides and numbers detected by telemetry at the monitored sites followed very similar trends at both daily and monthly scales. The high *R^2^* value indicated a strong positive relationship between the two metrics, with increases of daily relative abundance of grey reef sharks observed by dive guides matched by a corresponding linear increase in numbers of grey reef sharks detected by telemetry. Data generated by professional dive guides have great potential for providing estimates of relative abundance [15], [16], changes in population size over small and large scales [7], [19], [31] and insights into the ecology and population trends of marine predators in reef and coastal habitats [15], [20] however, the biases and limitations of such datasets remain poorly understood. One earlier study found that experience levels of observers made little difference in their abilities to detect sharks [7]. However, another study suggested that observations by dive guides might underestimate site fidelity [32]. Our study is the first to examine the ability of experienced observers to monitor patterns at a variety of temporal scales. For the most part, we found that dive guides produced datasets of shark relative abundance that tightly mirrored patterns generated by acoustic telemetry. Indeed, in some circumstances counts by dive guides may have been more accurate than those obtained by telemetry techniques. For example, one of the few discrepancies between dive guide observations and telemetry dataset occurred between March and April, when the relative proportion of sharks detected by telemetry was lower than those recorded by dive guides. This result might be a consequence of the presence of transient sharks during these months or higher attendance of individuals not tagged but that frequently visited the monitoring sites during this period. However, lower values of telemetry more likely reflected a decrease in receiver efficiency [33], since the timing coincided with field work in Palau when new tags were deployed on sharks. In support of this, an analysis of receiver metrics showed that signal collisions resulting from the large number of tags in the vicinity of some receivers at this time undermined the performance of the array (see [26] for more detail).

Although there was a close correlation between dive guide counts and telemetry results, to some extent this may have been a function of the particular circumstances of our study. We used experienced dive guides to gather data and the addition of tourist divers and less experienced guides might have reduced the strength of the relationship. While there is some evidence that diving experience may not necessarily be positively correlated with count accuracy [7], this is likely to depend on the circumstances surrounding the dive and may only be the case under relatively benign conditions of low current, simple topography and with relatively few sharks. During the study, experienced guides leading groups of tourist divers tended to follow predetermined routines, visiting specific landmarks over a time bounded by limits for safe recreational diving. Given the perpetually clear waters on the outer reefs of Palau (visibility typically >30 m), this meant that the sampling area covered by guides was likely to remain relatively constant among dives at a given dive site. The opportunity to view sharks is a focal point of the diving tourist experience in Palau [4], so that dive guides are likely to actively locate sharks during a dive. Additionally, dive guides are familiar with the local fauna at each dive site, which is also likely to reduce misidentifications and search effort. Finally, the sharks in many of the popular dive sites appear to be uninterested in, and relatively unwary of, divers. Indeed, we found no significant correlation between the numbers or depth of sharks recorded by telemetry and the numbers of tourist divers present in the dives. The rapid habituation of sharks to the presence of divers was first noted in some of the early behavioural studies in reef systems [34]. At our study sites, this behaviour meant that sharks were likely to remain in the local area despite the presence of tourist divers, making it relatively easy to obtain reliable counts of numbers. Together, these characteristics of the diving experience in Palau mean that it is ideally suited for a citizen science approach to shark monitoring in partnership with the recreational diving community. While the degree to which such features exist at other recreational diving localities in the wider Indo-Pacific region is unclear, the broad distribution of the species monitored in our study (grey reef and whitetip reef sharks) and the generally favourable diving conditions on coral reefs suggest that similar monitoring programs could be implemented in many locations across the region.

To be successful, monitoring programs need to have well defined objectives and standardised protocols that are effective in collecting accurate data of the target species. In our study, this was possible through data collection by recreational dive guides. However, alternative strategies might be necessary for species of sharks where diving conditions do not allow for underwater visual surveys by recreational divers, such as turbid coastal waters or waters below the limits of recreational diving depths. For such areas, programs designed to collect information from recreational catch and release fishers [35] and visual census conducted from vantage points [36] could provide useful citizen science data for the monitoring of shark populations.

Our study suggests that programs that use dive guides to monitor shark relative abundance in coral reefs could be a cost-effective alternative to traditional science-based surveys. However, this is not to imply that the set up and maintenance of such monitoring programs do not involve considerable logistics. We found that the success of our long-term program relied in part on sufficient resources for personnel, training and management of datasets to ensure data quality. On-ground leadership, encouragement and feedback was required to ensure that participants remained engaged in the program and maintained regular sampling throughout the study, as has been highlighted in other studies [16]. In part, this was done through incentive schemes by the dive tourism operator that involved small rewards (donated by local industry) to those guides that provided the most regular returns of questionnaires on an annual basis.

### Environmental Influences on the Abundance Patterns of Sharks

Dive guide data revealed a seasonal cycle in relative abundance of sharks, with peaks from March to June and the lowest values recorded in October and November. Dive guides also recorded relatively low numbers of both grey and whitetip reef sharks in May, a pattern that was more pronounced for the former species. This decrease in relative abundance coincided with an increase in water temperatures from around 25°C in previous months to a peak of 29°C in May [4]. Reproduction in reef sharks is known to be closely tied to temperature variation [37] and it may be that this sudden decline in the numbers of sharks may result from reproductive events occurring elsewhere on the reef that coincided with the peak in water temperatures. Some support for this hypothesis is found in dive guide observations of numerous females with fresh mating scars at this time.

Short-term (daily, weekly) changes in relative abundance of sharks at the monitored sites were correlated with current strength and temperature. Current strength appeared to be the more important driver of variations in relative abundance of whitetip reef sharks, with mean relative abundance steadily increasing with the current flow. For grey reef sharks, our models identified a combination of both current strength and temperature as the principal drivers of relative abundance. These sharks were three times more abundant during dives when currents were strong and relative abundance was also higher when water temperatures decreased from 30°C to 28°C. Earlier studies have noted the association of reef sharks with areas of strong current flow, typically around reef promontories, channels and passes [38], although why this occurs remains unclear. Our previous analyses of telemetry data at the same sites in Palau [26] also show that water temperature strongly influences the vertical movements of reef sharks, so that the mean depths occupied by sharks are greater when the layer of warm water near the surface (<40 m depth) expands to deeper waters (>60 m depth) [26]. Therefore, the reduction in number of sharks observed by dive guides during times of higher water temperatures could be associated with sharks occupying a greater vertical range of habitat, much of which is inaccessible to most recreational divers (i.e. >40 m depth) during these periods.

## Conclusions

Some scepticism surrounds the use of citizen science due to potential problems with the quality of data generated by untrained observers [8]. While mindful of the caveats mentioned above, we showed that counts by dive guides in Palau provided an effective method to monitor shark relative abundance. Our approach is relevant to many other citizen science initiatives because the technique of acoustic telemetry that we used to validate data collection by dive guides is one of the most rapidly-growing means of monitoring animals in marine environments. For example, collaborative initiatives such as the Ocean Tracking Network have now deployed acoustic arrays for tracking whales, seals, sharks, penguins, fish and a huge range other species in marine environments worldwide (see: http://oceantrackingnetwork.org/about/ocean). The development of arrays of listening stations by programs such as these offers the opportunity to validate citizen science initiatives, since both often target the charismatic megafauna that inhabit coastal systems. Such comparative studies will be necessary to ensure that the data that citizen science initiatives provide to management and conservation strategies in marine systems is credible, precise and reliable.
